# A digital score of peri‐epithelial lymphocytic activity predicts malignant transformation in oral epithelial dysplasia

**DOI:** 10.1002/path.6094

**Published:** 2023-06-09

**Authors:** Raja Muhammad Saad Bashir, Adam J Shephard, Hanya Mahmood, Neda Azarmehr, Shan E Ahmed Raza, Syed Ali Khurram, Nasir M Rajpoot

**Affiliations:** ^1^ Tissue Image Analytics Centre, Department of Computer Science University of Warwick Coventry UK; ^2^ Academic Unit of Oral & Maxillofacial Surgery, School of Clinical Dentistry University of Sheffield Sheffield UK; ^3^ Unit of Oral & Maxillofacial Pathology, School of Clinical Dentistry University of Sheffield Sheffield UK

**Keywords:** oral dysplasia, oral precancer, oral cancer, malignant transformation, computational pathology, deep learning, artificial intelligence, early detection

## Abstract

Oral squamous cell carcinoma (OSCC) is amongst the most common cancers, with more than 377,000 new cases worldwide each year. OSCC prognosis remains poor, related to cancer presentation at a late stage, indicating the need for early detection to improve patient prognosis. OSCC is often preceded by a premalignant state known as oral epithelial dysplasia (OED), which is diagnosed and graded using subjective histological criteria leading to variability and prognostic unreliability. In this work, we propose a deep learning approach for the development of prognostic models for malignant transformation and their association with clinical outcomes in histology whole slide images (WSIs) of OED tissue sections. We train a weakly supervised method on OED cases (*n* = 137) with malignant transformation (*n* = 50) and mean malignant transformation time of 6.51 years (±5.35 SD). Stratified five‐fold cross‐validation achieved an average area under the receiver‐operator characteristic curve (AUROC) of 0.78 for predicting malignant transformation in OED. Hotspot analysis revealed various features of nuclei in the epithelium and peri‐epithelial tissue to be significant prognostic factors for malignant transformation, including the count of peri‐epithelial lymphocytes (PELs) (*p* < 0.05), epithelial layer nuclei count (NC) (*p* < 0.05), and basal layer NC (*p* < 0.05). Progression‐free survival (PFS) using the epithelial layer NC (*p* < 0.05, C‐index = 0.73), basal layer NC (*p* < 0.05, C‐index = 0.70), and PELs count (*p* < 0.05, C‐index = 0.73) all showed association of these features with a high risk of malignant transformation in our univariate analysis. Our work shows the application of deep learning for the prognostication and prediction of PFS of OED for the first time and offers potential to aid patient management. Further evaluation and testing on multi‐centre data is required for validation and translation to clinical practice. © 2023 The Authors. *The Journal of Pathology* published by John Wiley & Sons Ltd on behalf of The Pathological Society of Great Britain and Ireland.

## Introduction

Oral cancer is amongst the most common cancers in the world and is considered a major health problem due to its significant associated morbidity and mortality [1]. The 5‐year survival rate has not improved over the last few decades regardless of improvements in surgical and oncological treatments. A large majority of oral cancers (>90%) are oral squamous cell carcinoma (OSCC), with one of the biggest obstacles to improvement in prognosis being the delayed presentation of disease, as evidenced by the fact that survival for stage I OSCC is 80%, which reduces to 20–30% for stage IV disease [2, 3]. OSCC is caused by a multitude of genetic and environmental factors and is preceded in a majority of cases by a potentially malignant state with proliferation of atypical epithelium known as oral epithelial dysplasia (OED) [4]. Dysplastic lesions have been shown to have an increased risk of progression to malignant transformation [5]. Unfortunately, at present, there are no specific clinical tools or biological or molecular markers routinely used or recommended in clinical practice for prognosticating dysplastic lesions. Some clinical risk predictors have been suggested to be helpful, including size, clinical site (e.g. floor of mouth, lower gums, lateral tongue), and clinical appearance (e.g. leukoplakia, erythroplakia) and can be found in a wide range of conditions collectively referred to as oral potentially malignant disorders (OPMDs) in clinical practice [6].

In practice, OED diagnosis and grading are performed on a tissue biopsy using histological assessment and light microscopy. The current gold standard grading system (the 2017 WHO grading system that uses three tiers of grading: mild, moderate, or severe dysplasia) is subjective, taking into account more than 15 different cytological and architectural features to determine the OED grade, which guides treatment decisions [4]. However, the cytological and architectural features are ill defined and lacking in prognostic value, e.g. mild or moderate OED can progress to malignancy, whereas severe OED may not [6]. In addition, OED grading suffers from significant inter‐ and intra‐observer variation due to its subjective nature, and interpretation can be hugely dependent upon the observer's experience and training. To improve diagnostic reproducibility and prognostication, Kujan *et al*. [7] introduced the idea of a binary grading system, categorising cases as either low or high risk depending on the number of architectural and cytological features seen. Although reports have suggested improvement of diagnostic agreement and prognosis using the binary system, it also has shortcomings and has not been widely adapted for clinical use, highlighting the need for novel approaches [6, 8] bringing objectivity and better prognostic value to inform patient management and aid treatment decisions [9].

With wider adaptation of digital pathology in clinical practice, artificial intelligence (AI) algorithms have also evolved and have shown promise for automated detection and quantification of histological features for classification [10, 11, 12, 13, 14], detection [15, 16, 17, 18], segmentation [19, 20, 21], and survival analysis [18, 22]. Digitisation of histology slides along with AI can be used to develop algorithms to assist pathologists in diagnostic decision‐making and lead to better prognostication for improved patient management. To the best of our knowledge, there has been limited research on the computational analysis of OED histology images for the prediction of malignant transformation. Existing methods in the literature have used relatively small cohorts, manual elements, or region of interest (ROI)‐based analyses [14, 23, 24, 25, 26, 27, 28, 29, 30]. All these methods have focused mainly on OED identification or grading and lack predictive or prognostic ability. Limited computational pathology work has been reported at the WSI level for the predictive analysis of OED, including recurrence and malignant transformation potential. Dost *et al*. [30] examined 368 OED patients where 7.1% progressed to carcinoma and showed that there was no association of OED grade with malignant transformation. Gilvetti *et al*. [31] reported a study including 120 patients with mean follow‐up of 47.7 months (±29.9 SD) and showed that recurrence rate was significant in high‐grade OED patients with erythroplakia, with *p* = 0.023 and mean time to recurrence of 62 months (±31.5 SD). Malignant transformation was also shown to have significant association with age (*p* = 0.034), clinical appearance (*p* = 0.030), lesion site (*P* = 0.007) and some other clinical features with mean transformation time of 50 months (±32.5 SD). A recent study by Mahmood *et al*. [32] examined the correlations between individual histological features and OED prognosis. They examined OED biopsies from 108 patients with a minimum of 5‐year follow‐up to analyse histological features predictive of recurrence and malignant transformation. Two different prognostic models based on the presence of specific histological features (bulbous rete processes, hyperchromatism, loss of epithelial cohesion, loss of stratification, suprabasal mitoses, and nuclear pleomorphism, irrespective of grade) were proposed, with an area under the receiver‐operator characteristic curve (AUROC) value of 0.77 for malignant transformation and 0.72 for recurrence. This highlights the usefulness of individual (grade‐independent) histological features for OED prognosis prediction. A significant proportion of OED lesions can transform into malignancy (OSCC), but at present there are no tools available for an objective and reproducible prediction of malignant transformation. Early prediction of malignant transformation is crucial to aid patient care and inform appropriate treatment to improve prognosis and reduce the need for radical and disfiguring surgery later.

In this study, we investigated the effectiveness of deep learning algorithms for prognostication from haematoxylin and eosin (H&E) stained WSIs.

## Materials and methods

### Data

The dataset used for this study comprised 163 H&E stained and scanned WSIs of control and OED cases between 2005 to 2016. WSIs were scanned at ×20 magnification using an Aperio CS2 scanner (Leica Biosystems, Deer Park, Illinois, USA) (*n* = 66) and at ×40 using a Hamamatsu Nanozoomer scanner (Hamamatsu City, Shizuoka Prefecture, Japan) (*n* = 97) after ethical approval (REC Reference18/WM/0335, National Health Service Health Research Authority West Midlands). Amongst 163 cases, 137 were OED cases with 50 transformed into malignancy. The remaining cases were non‐dysplastic oral mucosal biopsies, including benign hyperkeratosis or mild epithelial hyperplasia. The mean age in the dataset of OED cases was 64.64 (range 25–97) with a mean age of men (*n* = 84) of 66.3 and mean age of women (*n* = 53) of 64.5 The main clinical sites of involvement were the tongue, floor of mouth, and buccal mucosa. The mean time to malignant transformation was 6.51 years (±5.35 SD).

WSIs were included if the following conditions were met: a histological diagnosis of OED, sufficient availability of tissue (i.e. excluding tangentially cut sections, tissue with artefacts), at least 5 years of follow‐up data (including treatment, recurrence, and transformation information) from the initial diagnosis, and all cases were independently seen by at least two certified/consultant pathologists. The initial diagnoses were made by PMS, PMF, and DJB as part of their diagnostic workload (see Acknowledgements) using the WHO grading system. These cases were blindly seen/reported/graded by SAK.

The interobserver disagreement between the two pathologists was assessed using Cohen's kappa score, which resulted in a value of 0.854. The score indicates a high level of agreement between the two pathologists. Cases with disagreement were resolved through discussion within the team. More information about the cohort is presented in Table 1. Epithelium masks were obtained using HoVer‐Net+ [9] and then refined manually for some cases, whereas slide‐level labels were obtained for each case from patient records (i.e. clinical notes and biopsies), including histological grades, recurrence status, and malignant transformation status (i.e. OED has progressed into OSCC at the same diagnosed location within the follow‐up time). The WSIs were split into training and testing sets using three different stratified five‐fold cross‐validations on transformation status for all experiments. Patches with dimensions 512×512 were extracted using the epithelium mask with an overlap of 50% from all the WSIs at 0.50 μm per pixel (mpp). To extract the deep features, ResNet‐50 [33] was used as a feature extractor pretrained on ImageNet. A feature vector of size 1,024 was extracted for each patch, resulting in a bag of shape $x\in {\mathbb{R}}^{n\times 1024}$ for all WSIs (where n is the number of patches extracted).

**Table 1:** Characteristics of cohort used in study with clinical and demographic information on OED cases.

Characteristic	Number (%)
OED cases	137
Cases with malignant transformation	50 (36.4%)
WHO grade	
Mild	41 (29.9%)
Moderate	53 (38.6%)
Severe	43 (31.3%)
Binary grade	
Low risk	80 (58.3%)
High risk	57 (41.6%)
Mean age [min–max]	64.64 [25–97]
Gender	
Male	84 (61.3%)
Female	53 (38.6%)
Clinical (intra‐oral) site	
Tongue	53 (38.6%)
Floor of mouth	27 (19.7%)
Buccal mucosa	17 (12.4%)
Others	38 (27.7%)

Survival	Mean (SD)
Survival (months)	84.75 (63.03)
Survival (years)	6.51 (5.35)

### Malignant transformation prediction

**Figure 1:**
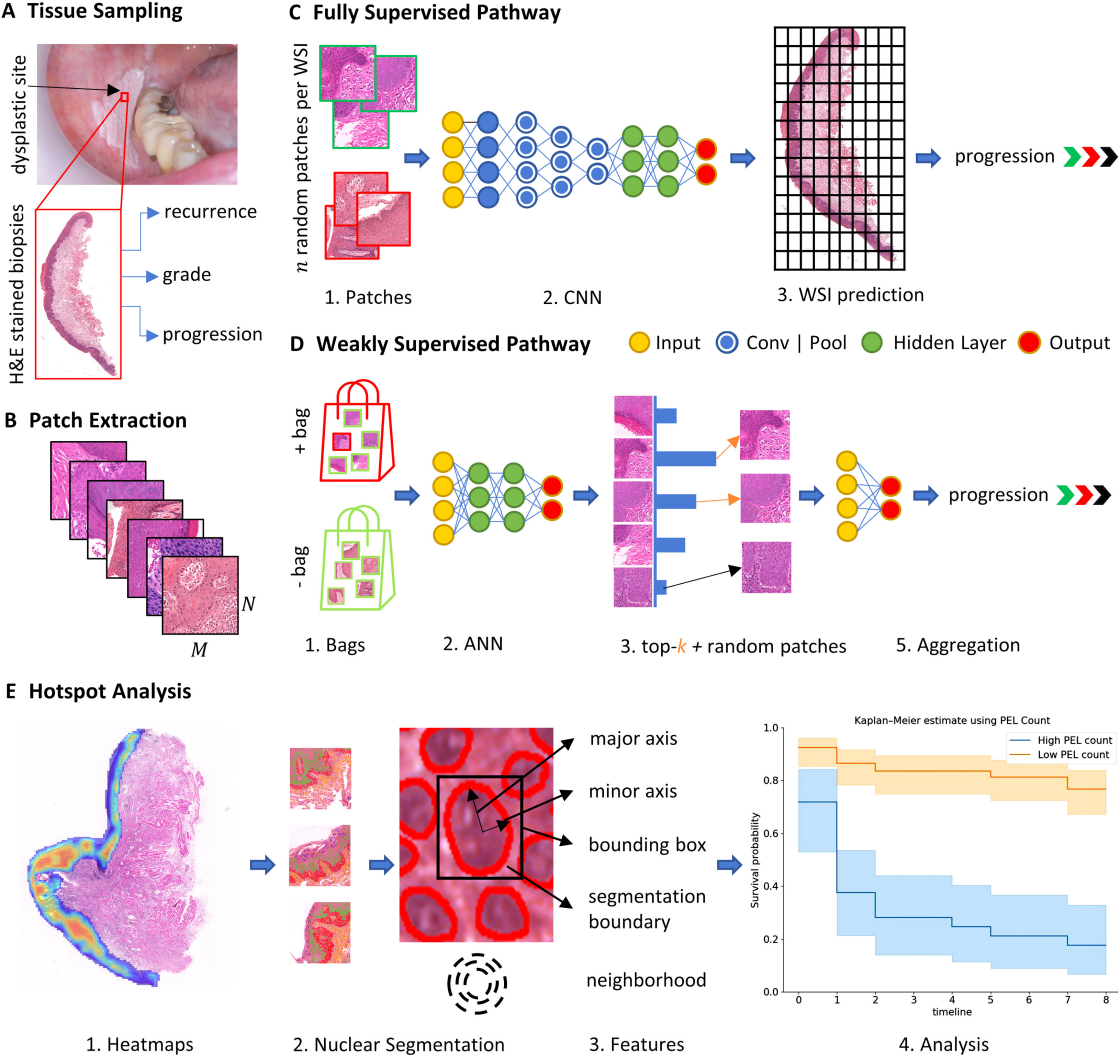
Overall workflow of the study is shown in different sections. (A) Process of obtaining tissue biopsies from dysplastic lesions and corresponding WSIs with their associated labels assigned by a pathologist. (B) Patches of size *M × N* were extracted from epithelium region of WSIs. (C) Fully supervised pipeline where patches were assigned the WSI level labels and trained using CNNs for downstream tasks. (D) Weakly supervised pipeline where positive (+) and negative (−) batch of features/images was created and used for training. (E) Heatmaps were generated using IDaRS to explore hotspot areas and their contribution to malignant transformation prediction using nuclear analysis. Nuclear features from hotspots and coldspots were used for PFS.

Figure 1 shows the overall pipeline, which involves initially extracting X patches of size M×N with slide‐level labels Y from WSIs with an overlap of O using the epithelium mask. Extracted patches were utilised for training deep learning models for predicting malignant transformation. In this study, we used iterative draw‐and‐rank sampling (IDaRS) [34], which works by ranking and selecting top and random patches from a WSI assuming that not all patches are equally important and predictive of the outcome. IDaRS selects two subsets of patches for training including random patches r and top‐ranked patches k for each WSI. Both subsets are then preprocessed using a standard set of augmentations and train a convolutional neural network (CNN) with weak labels. We also compared the IDaRS with other fully supervised and weakly supervised algorithms, for example multilayer perceptron (MLP), Attention‐MIL (A‐MIL) [35], clustering constrained attention multiple instance leaning (CLAM) [36], and CNN‐based benchmark classification models (ResNet [33], DenseNet [37], and Vision Transformers [38] with max pooling as an aggregator for the final WSI label).

### Cellular composition analysis

To further analyse and validate hotspots being identified by the IDaRS model cellular compositions of top tiles (i.e. hotspots and coldspots) from transformed and non‐transformed cases were analysed. Nuclear features were extracted from each layer (i.e. keratin, epithelial, basal) and associated connective tissue in an automated manner using nuclear segmentation and classification. For this purpose, input patches were first stain‐normalised using a sample from The Cancer Genome Atlas (TCGA) cohort before being fed into HoVer‐Net [15], which was pretrained on the PanNuke dataset [21] for nuclear instance segmentation and classification. For the segmentation of the keratin, epithelial, and basal layers within the epithelium, HoVer‐Net+ [9] was used. Table 2 shows a range of morphological and proximity features extracted from the segmented image patches and aggregated statistically using the minimum ∧, maximum ∨, mean μ, median m, and standard deviation σ. Here, ordinary least squares (OLS) was used with *post hoc t*‐tests for calculating statistical significance with Benjamini–Hochberg adjustment [39]. Cellular composition helps in understanding/interpreting the results of IDaRS and differentiating transformed cases from non‐transformed ones in an objective manner.

**Table 2:** Nuclear features extracted from layer‐wise nuclei and their explanations.

Feature	Explanation
Extent $\left(\mathrm{EX}\right)$	Ratio of bounding box pixels to total region
Equivalent diameter $\left(\mathrm{ED}\right)$	Diameter of circle in bounding box
Eccentricity $\left(\mathrm{ECC}\right)$	Ratio of focal distance over major axis
Convex area $\left(\mathrm{CA}\right)$	Number of pixels in convex hull
Centroid (C)	Centre location of bounding box
Major axis length $\left(\mathrm{MJL}\right)$	Length of major axis
Nuclei count $\left(\mathrm{NC}\right)$	Total number of nuclei in patch
Cellularity per micron $\left(\phi \right)$	Nuclei density in patch per micron
Nearest‐neighbour distance (NND)	Nearest nucleus distance from nucleus of interest

### Peri‐epithelial lymphocyte count

Elevated peri‐epithelial lymphocyte (PELs) counts can be linked to a higher risk of malignant transformation in OED, and to further explore the role of PELs count in transformed and non‐transformed cases, a Wilcoxon rank‐sum test was performed, where *p* < 0.05 was considered significant. Moreover, we also analysed the distributions of PELs counts in subgroups based on two clinical features, gender and age. Gender was divided into male and female groups, and the age subgroups were separated into ranges of 0–50, 51–70, and 71–100.

### Survival analysis

To investigate the prognostic significance of clinical, pathological, and nuclear features for progression‐free survival (PFS), Kaplan–Meier (KM) curves and Cox proportional hazard (CPH) models were used for univariate and multivariate analysis. To distinguish between high‐risk (short‐term survival) and low‐risk (long‐term survival) groups, the optimal cut‐off value was calculated by taking the mean of the hazard value for each instance using a CPH model where the statistical significance was large between the high‐ and low‐risk groups. Further, a log‐rank test was performed to determine the statistical significance, with *p* < 0.05 considered statistically significant.

### Experiments

For IDaRS, we set the random patches r=30 and top patches k=5 and trained a pretrained ResNet‐34 on ImageNet with a batch size of 16 and input size of 256. IDaRS was trained for 30 epochs with binary cross‐entropy loss and optimised using the Adam optimiser. For training, MLP and CLAM deep features were then fed as input to models for generating WSI‐level outputs. MLP and CLAM were trained for 1,000 epochs using the default configurations from the CLAM [36]. For A‐MIL and CNN models the same input and configurations as IDaRS were used for the training and test purposes. All models were trained and tested on a system with two Nvidia Titan‐X GPUs with 12 GB memory, dedicated RAM of 128 GB, and an Intel® Core i9 processor, where an average epoch takes ~10 min and downstream analysis for a single WSI takes ~1 min.

To validate the results, stratified on transformation status, five‐fold cross validation was performed three times with different random seeds. AUROC and F1 score (macro) were used as performance metrics and are averaged across the folds. The F1 score can be thought of as the weighted mean between the precision and recall as

$$F1=\frac{2\times \left(\text{Precision}\;\times \;\text{Recall}\right)}{\left(\text{Precision}\;+\;\text{Recall}\right)}.$$

The F1 score (macro) computes the arithmetic mean of the F1 score per class, treating all classes equally regardless of their number. AUROC evaluates the binary problems by plotting the true positive rate (TPR) against the false positive rate (FPR) at various thresholds. It measures the ability of the classifier to differentiate between the two classes, where the TPR and FPR are calculated as

$$\mathrm{TPR}=\frac{\text{True positives}}{\text{True positives}\;+\;\text{False negatives}},$$

$$\mathrm{FPR}=\frac{\text{False positives}}{\text{False positives}\;+\;\text{True negatives}}.$$

## Results

### Malignant transformation

**Figure 2:**
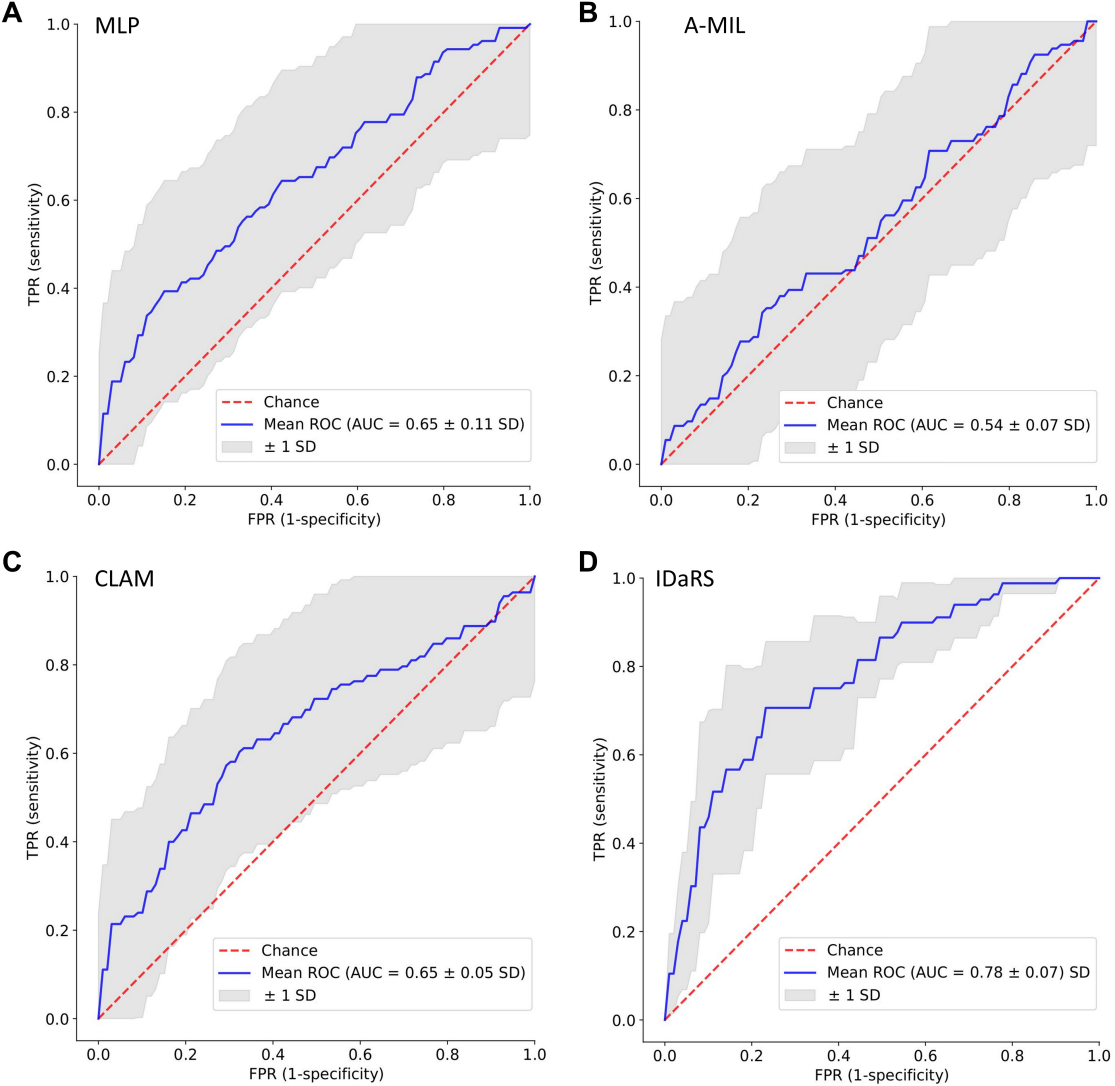
ROC curve plots on five‐fold cross validation for OED malignant transformation prediction using (A) MIL, (B) A‐MIL, (C) CLAM, and (D) IDaRS.

Our experiments, summarised in Table 3, indicate that the performance of IDaRS is comparatively better than that of other weakly and fully supervised algorithms with an AUROC of 0.78 (±0.07 SD) and F1 score of 0.69 (±0.05 SD) compared to MLP, CLAM, and A‐MIL. It can also be observed from the ROC plots in Figure 2 that the standard deviation across different folds for IDaRS is smaller compared to the other weakly supervised algorithms. The performance of CLAM was competitive to IDaRS compared to the MIL in terms of the F1 score. The fully supervised networks performed worse than other weakly supervised models due to the inherent nature of the problem, which introduces noise in the labels and corrupts the model's training.

**Table 3:** Performance of IDaRS model compared to other weakly supervised and fully supervised models with deep features where IDaRS achieves high performance in terms of AUROC.

Model	Top‐k	AUC ± SD	F1 score ± SD
MLP	1	0.65 ± 0.09	0.56 ± 0.11
	5	0.64 ± 0.11	0.55 ± 0.01
Attention‐MIL [35]	‐	0.54 ± 0.07	0.44 ± 0.03
CLAM [36]	1	0.65 ± 0.04	0.64 ± 0.04
	5	0.65 ± 0.05	0.63 ± 0.01
IDaRS [34]	5	0.78 ± 0.07	0.69 ± 0.05
ResNet‐50 [33]	‐	0.54 ± 0.10	0.43 ± 0.11
ViT [38]	‐	0.55 ± 0.01	0.45 ± 0.08
DenseNet [37]	‐	0.56 ± 0.05	0.44 ± 0.01

### Exploring visual patterns

**Figure 3:**
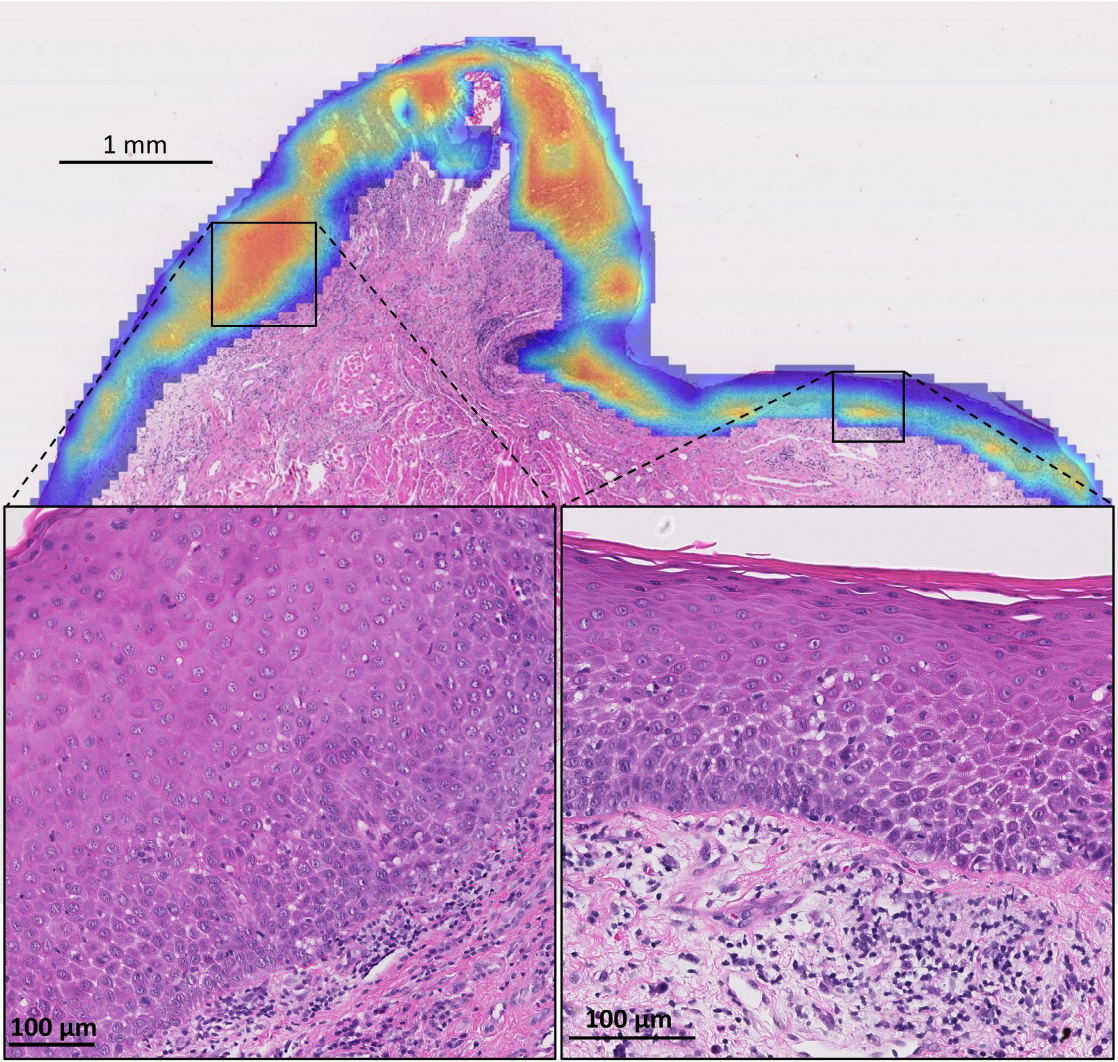
Heatmap for malignant transformation prediction using IDaRS. The red region shows the high probability of malignant transformation in those areas.

To investigate the features learnt by the top performing IDaRS, we explored the top tiles from the heatmaps of the transformed and non‐transformed WSIs. To correlate hotspot/coldspots with the clinical features, heatmaps were also analysed manually for corroboration purposes by an expert pathologist (SAK). Figure 3 shows the heatmap for a histologically high‐risk case where red (hotspot) represents a region with a higher probability of malignant transformation, while blue (coldspot) corresponds to a region with a low probability of transformation. Closer examination of hotpots shows evidence of disordered stratification, dyskeratosis, and nuclear and cellular pleomorphism with a dense lymphocytic infiltrate in the adjacent peri‐epithelial connective tissue. The dense lymphocytic infiltrate is referred to as PELs for the remainder of the analysis.

### Cellular composition analysis

Following the manual analysis of the heatmaps, automated cellular composition analysis was performed to uncover significant hidden patterns/features in transformed versus non‐transformed cases. Table 4 shows the prognostic significance of the extracted nuclear features for predicting malignant transformation. For the epithelial layer, variation in eccentricity (*p* = 0.048), bounding box (*p* = 0.0487), and total nuclei count (*p* < 0.0001) showed significance along with basal layer NC (*p* < 0.0001). An increase in cell count (hyperplasia or crowding) is an important feature observed in high‐risk dysplasia in both the central epithelium layer and specifically within the basal layer. Other features in the epithelium, for example variation in nuclei count (100 μm per pixel) and nearest nucleus distance, correspond to congestion in the spatial arrangements of the epithelial nuclei and require more data for validation. Similarly, changes in basal layer nuclei's minor axis and equivalent diameter correspond to nuclear pleomorphism and are observed in high‐risk OED cases. Interestingly, the nuclei count in the connective tissue area also showed significance for predicting the transformation (*p* = 0.0004), which corresponds to the previous observation regarding the dense lymphocytic infiltrate in the adjacent peri‐epithelial connective tissue.

**Table 4:** Ordinary least‐squares regression for malignant transformation with *t*‐test significance of nuclear features with Benjamini–Hochberg [39] adjustment.

Feature	*P* > |*t*|	*P* > |*t*| (adjusted)
Tissue NC	0.0013	0.0481*
Tissue σ nuclei in 100 mpp	0.0289	0.2755
Tissue max eccentricity	0.0428	0.3491
Basal μ minor axis length	0.0436	0.3491
Basal σ ED	0.0090	0.1672
Basal NC	<0.0001	<0.0001*
Epithelium μ eccentricity	0.0015	0.0487*
Epithelium μ NND	0.0099	0.1672
Epithelium μ nuclei in 100 mpp	0.0125	0.1273
Epithelium σ eccentricity	0.0028	0.0729
Epithelium σ bounding box	0.0010	0.0487*
Epithelium NC	<0.0001	<0.0001*

Asterisk (*) indicates significant *p* value. σ = SD, μ = mean of a distribution.

### Peri‐epithelial lymphocytes

**Figure 4:**
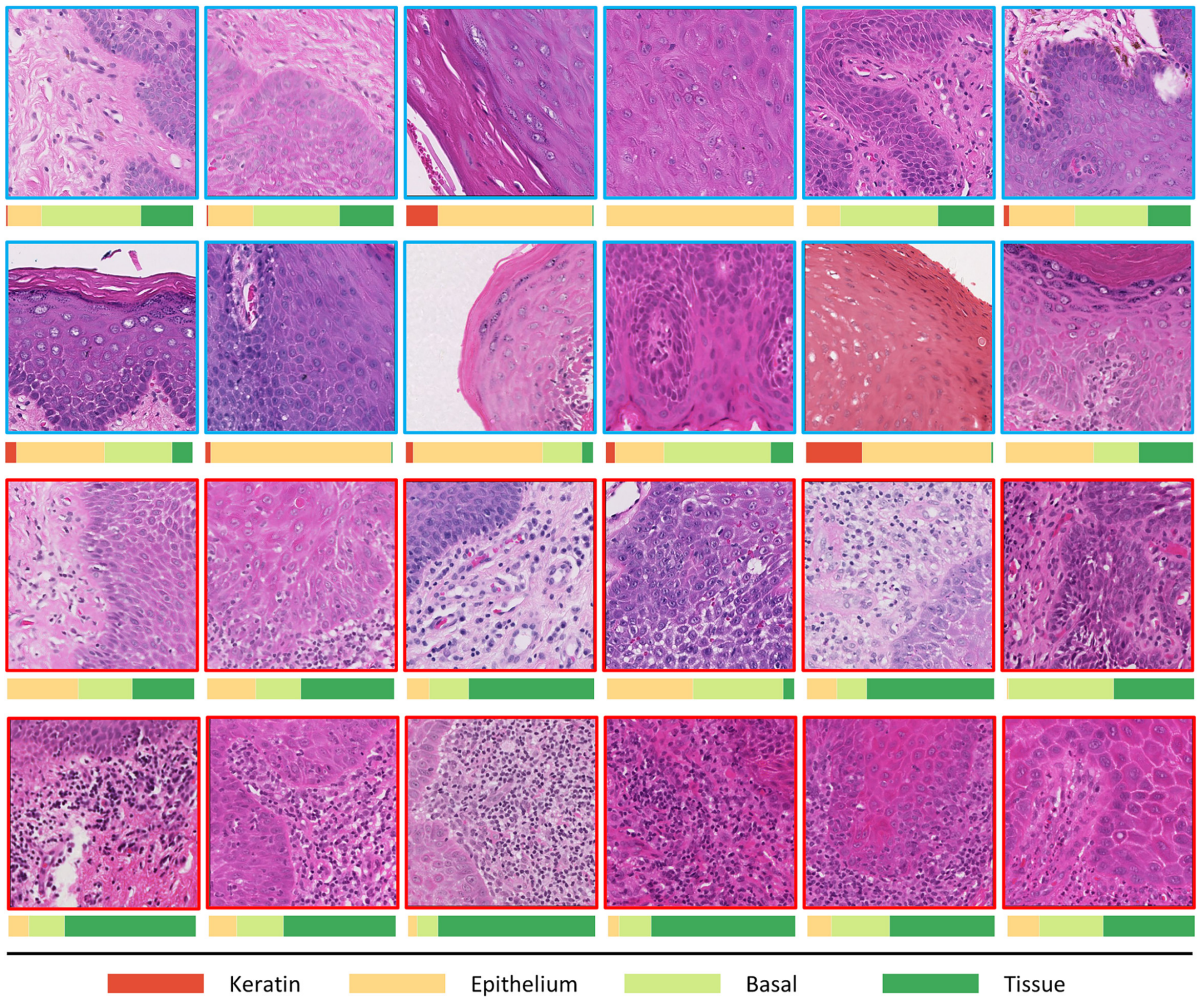
Patches extracted from hotspots (red) and coldspots (blue) of WSIs with their layer‐wise nuclear composition. Most coldspot regions have dominant epithelial nuclei compared to hotspots, where PELs can be seen dominating the overall ratio.

**Figure 5:**
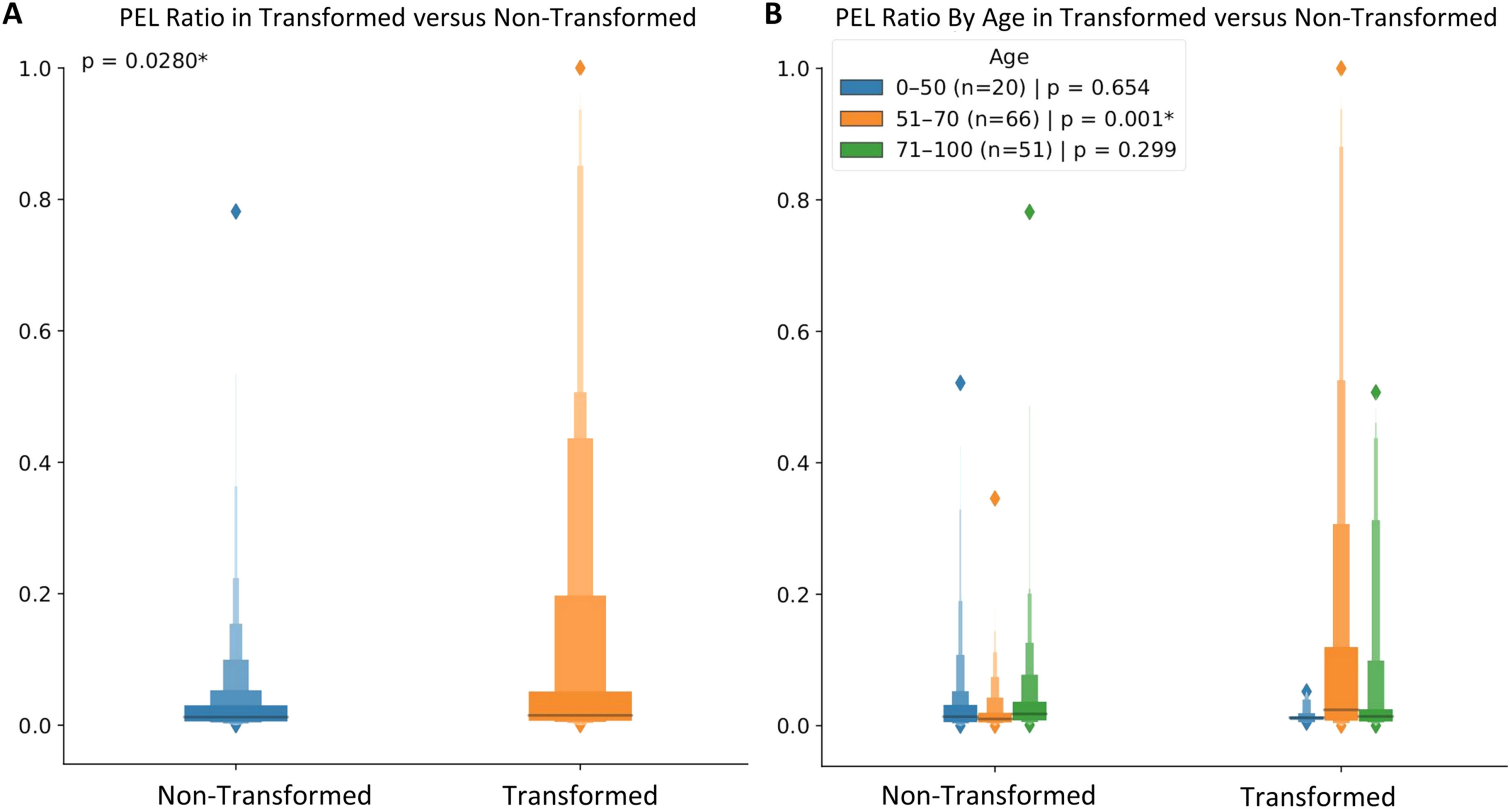
(A) Boxenplot for ratio of PELs present in both transformed and non‐transformed patches. (B) Further breakdown of PELs ratio into age groups, where it can be seen that the 0–50 age group has a distinct difference in the PELs ratio compared to other groups. Asterisk (*) indicates significant *p* value.

Figure 4 shows example patches from both hotspot (red) and coldspot (blue) regions of the transformed and non‐transformed cases with their corresponding layer‐wise cellular compositions. For most of the coldspots, the epithelium and basal nuclei are dominant, whereas in the hotspots (red), PELs are in abundance in the transformed cases compared to non‐transformed cases (Figure 4). PELs were statistically significant (*p* = 0.02) for differentiating between the transformed versus non‐transformed cases. Gender‐based subgrouping showed no significance between male and female groups. However, for age, the 0–50 group showed prognostic significance with respect to malignant transformation with *p* = 0.001. Figure 5 shows the boxen plots for (A) the overall distribution of PELs ratios in transformed cases versus non‐transformed cases and (B) the distribution of PELs ratios in transformed cases versus non‐transformed cases including age subgrouping.

### Survival analysis

**Figure 6:**
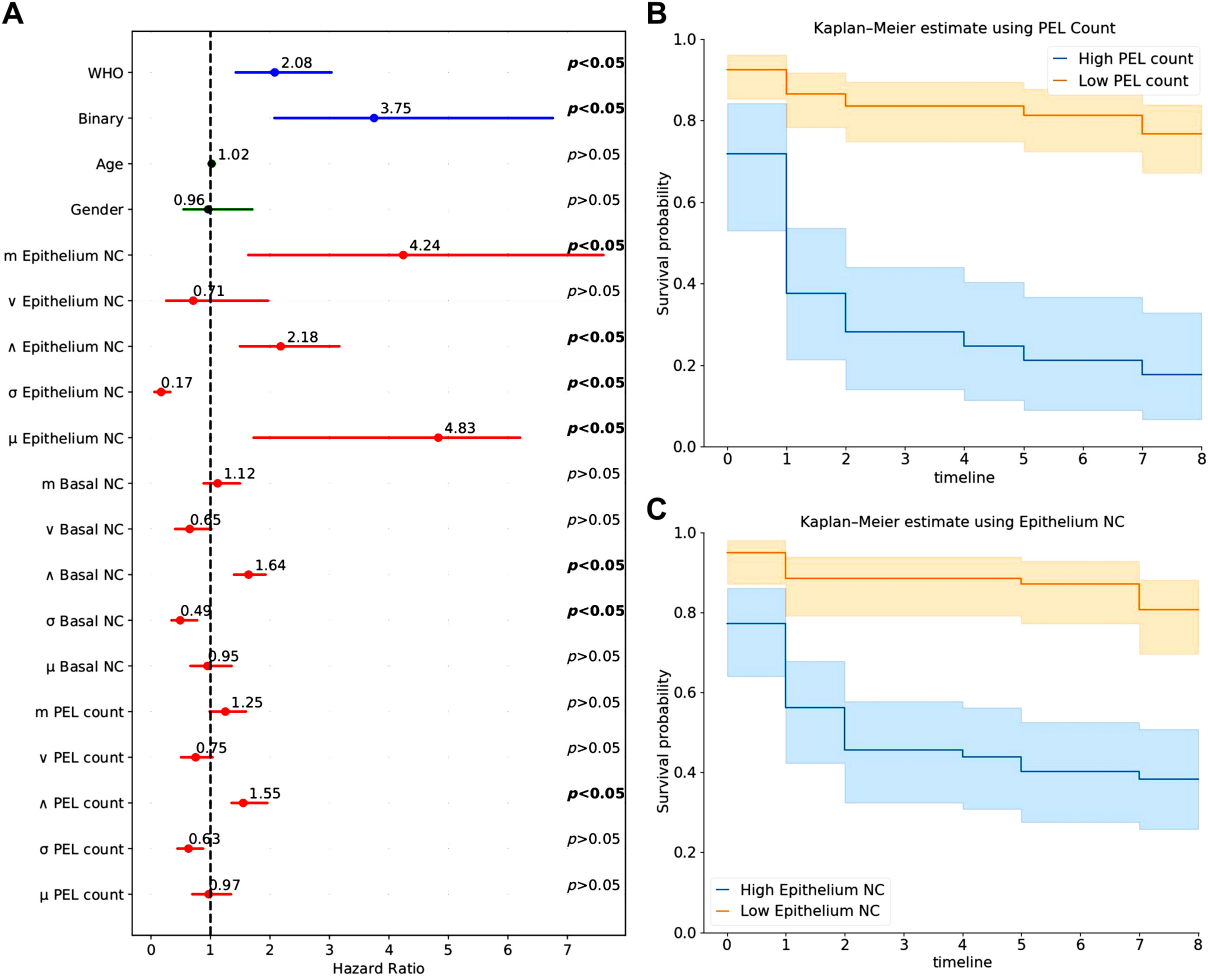
(A) Univariate analysis of different features: (blue) pathological, (green) clinical, and (red) nuclear. For each feature the dot represents the hazard ratio, and the filled line shows the lower and upper confidence intervals of 95%. *P* values are at right, calculated using Wald test, where significant *P* values are in bold. (B) Kaplan–Meir curve for PFS of OED using PELs count. (C) KM curve using epithelium layer nuclei count.

Table 5 shows the univariate analysis of the aforementioned nuclear features mentioned in ‘Cellular composition analysis' section, with clinical and pathological features, where it can be seen that the clinical features, age [*p* > 0.05, C‐index = 0.59 (95%, 0.59–0.60)] and gender [*p* > 0.05, C‐index = 0.52 (95%, 0.52–0.53)], are nonsignificant. Conversely, the pathological features showed significance for binary grading [*p* = 0.004, C‐index = 0.68 (95%, 0.67–0.69)] and WHO‐based grading when moderate and severe cases were combined against mild grade [*p* = 0.04, C‐index = 0.68 (95%, 0.67–0.68)]. When mild and moderate cases were combined and compared with severe cases, they showed the same significance [*p* = 0.04, C‐index = 0.68 (95%, 0.67–0.68)]. The nuclear features extracted from the epithelial layer, basal layer, and connective tissue area also showed significance for minimum number of nuclei count (NC) in basal layer [*p* < 0.05, C‐index = 0.70 (95%, 0.69–0.71)], epithelial layer [*p* < 0.05, C‐index = 0.73 (95%, 0.73–0.74)], and in PELs [*p* < 0.05, C‐index = 0.73 (95%, 0.72–0.73)]. Figure 6B shows the KM curves for PELs count and Figure 6C the epithelium layer NC, where both features are statistically significant in differentiating high‐risk and low‐risk lesions with a clear separation between two groups. Figure 6A shows the hazard ratio (HR) for variation in basal layer NC, and the epithelium layer NC appears to be associated with improved survival, whereas the minimum PELs count, epithelium layer NC, and basal layer NC are predictors of adverse PFS.

**Table 5:** Univariate analysis of clinical, pathological, and digital parameters where *P* is calculated using log‐rank method and C‐index is calculated using Cox proportional hazard model bootstrapped 1,000 times for lower and upper confidence interval.

Feature	Aggregation	*p*	C‐index	Lower 95%	Upper 95%
Clinical parameters					
Gender	‐	>0.05	0.52	0.52	0.53
Age	‐	>0.05	0.59	0.59	0.60
Pathological parameters					
WHO grading (mild versus mod + severe)	‐	<0.05	0.68	0.68	0.69
WHO grading (mild + mod versus severe)	‐	<0.05	0.68	0.68	0.68
Binary grading	‐	<0.05	0.68	0.68	0.69
Nuclear features					
PELs count	μ	>0.05	0.45	0.45	0.46
	σ	<0.05	0.60	0.59	0.60
	m	>0.05	0.57	0.56	0.58
	∧	<0.05	0.73	0.72	0.73
	∨	>0.05	0.53	0.52	0.54
Basal NC	μ	>0.05	0.45	0.44	0.46
	σ	<0.05	0.66	0.65	0.67
	m	>0.05	0.52	0.51	0.53
	∧	<0.05	0.70	0.69	0.71
	∨	>0.05	0.53	0.52	0.54
Epithelium NC	μ	<0.05	0.65	0.64	0.65
	σ	<0.05	0.72	0.71	0.73
	m	<0.05	0.66	0.65	0.67
	∧	<0.05	0.73	0.73	0.74
	∨	>0.05	0.46	0.45	0.47

σ = standard deviation, μ = mean, m = mode, ∧ = minimum, ∨ = maximum of a distribution.

Table 6 shows a multivariate analysis of the most significant nuclear and pathological features (i.e. binary grading, ∧ epithelial layer NC, ∧ basal layer NC, and ∧ PELs count) to examine their combined effect on PFS. When these features are combined, the C‐index improves, reaching 0.79 (95%, 0.78–0.80), with binary grading, epithelium layer NC, and PELs being the most significant prognostic features for malignant transformation. In the absence of binary grading, the C‐index achieved using nuclear features only is competitive, reaching 0.78 (95%, 0.77–0.78). Similarly, combined binary grading with PELs counts reached the same C‐index of 0.78 (95%, 0.77–0.78) compared to the other two feature with binary grading, i.e. epithelium layer NC 0.76 (95%, 0.75–0.77) and basal layer NC 0.77 (95%, 0.76–0.77). This highlights the importance of using PELs counts as a prognostic feature for predicting malignant transformation. Further, the combined performance of basal layer NC and epithelium layer NC with PELs count also shows the significance of using PELs in conjunction with other clinical and nuclear features.

**Table 6:** Multivariate analysis of pathological and digital features where *P* is calculated using Wald test and C‐index is calculated using Cox proportional hazard model bootstrapped 1,000 times for lower and upper confidence interval.

Feature	*P*	HR	Lower 95%	Upper 95%
C‐index = 0.79, 95% CI [0.78–0.80]				
Binary grading	<0.05	2.43	1.30	4.54
Basal NC	>0.05	1.04	0.79	1.37
PELs count	<0.05	1.72	1.24	2.37
Epithelium NC	<0.05	1.48	1.07	2.05
C‐index = 0.78, 95% CI [0.77–0.78]				
Basal NC	>0.05	1.08	0.81	1.43
PELs count	<0.05	1.72	1.23	2.39
Epithelium NC	<0.05	1.67	1.20	2.32
C‐index = 0.77, 95% CI [0.76–0.77]				
Binary grading	<0.05	2.97	1.62	5.43
Basal NC	<0.05	1.54	1.31	1.81
C‐index = 0.78, 95% CI [0.77–0.78]				
Binary grading	<0.05	3.10	1.70	5.65
PELs count	<0.05	1.81	1.50	2.18
C‐index = 0.76, 95% CI [0.75–0.77]				
Binary grading	<0.05	2.76	1.44	4.93
Epithelium NC	<0.05	1.84	1.27	2.66
C‐index = 0.73, 95% CI [0.72–0.74]				
Basal NC	>0.05	1.13	0.84	1.52
PELs count	<0.05	1.68	1.20	2.34
C‐index = 0.77, 95% CI [0.77–0.78]				
Epithelium NC	<0.05	1.67	1.19	2.35
Basal layer NC	<0.05	1.54	1.29	1.83
C‐index = 0.78, 95% CI [0.77–0.78]				
Epithelium NC	<0.05	1.68	1.21	2.34
PELs count	<0.05	1.83	1.50	2.25

## Discussion and conclusions

In this study, we explored the potential of deep learning for predicting malignant transformations from digitised OED histology slides. We trained a weakly supervised learning framework for malignant transformation prediction and further analysed the predictive ‘hotspots’ in epithelial and peri‐epithelial tissue regions. We demonstrated that deep learning‐based weakly supervised IDaRS could predict malignant transformation with an AUROC of ~0.78 (±0.07 SD) on stratified 5‐fold cross‐validation using three different random seeds. The higher performance of IDaRS compared to other MIL algorithms is explained by the fact that it dynamically learns important feature representations from patches internally, compared to fixed patch feature representation as an input limiting the learning possibilities of a model. Mahmood *et al*. [32] also reported an AUROC of 0.77 for transformation using a similar but smaller cohort with the nuclear features subjectively assessed by three pathologists.

We also explored the cellular compositions (i.e. nuclear features) and their role in potentially malignant areas (i.e. hotspots) of transformed cases and compared them to non‐transformed areas (i.e. coldspots). Nuclear features from the epithelial layer and associated connective tissue area were found to be the most significant prognostic features for predicting malignant transformation. Other important features found in the epithelial and basal layers were variation in the number of layer nuclei in 100 μm per pixel (mpp), standard deviation in cell eccentricity, and mean major and minor axis lengths, for example. These nuclear features also correspond to nuclear aberration (i.e. variation in size of nuclei captured as a variation in the minor axis of the nuclei and convexity of the nuclear shape) and congestion due to the proliferation of nuclei in the epithelial and basal layers. However, to verify the significance of these features, we require more data to test these features' ability to indicate prognostic significance for malignancy. It has also been reported in the literature that PELs can play an important role in transforming dysplasia into carcinoma [40]. There is a possible explanation for transformation, that the epithelium is affected by the PELs count. This can be due to the release of cytokines linked with oxidative stress, transforming the epithelial cells into premalignant ones [41, 42, 43, 44], and we have seen that PELs showed significance for predicting transformation with *p* < 0.05.

For PFS, we examined the clinical, pathological, and nuclear features of oral epithelial dysplasia. Our findings indicated that, in addition to binary grading, the variation in basal layer NC and epithelial layer NC was associated with improved PFS. On the other hand, we observed that the minimum number of nuclei in the basal layer, epithelial layer, and PELs were linked to a higher risk of malignant transformation or poor survival. Gan *et al*. [40] also investigated the potential role of lymphocytic infiltration in malignant transformation by analysing the RNA sequences of immune infiltration sites in moderate and severe OED. The authors highlighted the importance of immune signatures established from oral cancer to identify three distinct subtypes of moderate and severe OED: immune cytotoxic, non‐cytotoxic, and non‐immune reactive from transcriptional data. Their findings suggest that the lack of CD8 T‐cells in non‐cytotoxic subtype and non‐immune reactive subtype can lead to progression in moderate and severe dysplasia. In our study, we quantified the peri‐epithelial lymphocytes in the malignant transformed cases using deep learning. Our study identified binary grading as a significant indicator of malignant transformation in OED, whereas the study by Dost *et al*. [30] did not find any association between grading and transformation. However, Mahmood *et al*. [32] demonstrated an association between nuclear features used for OED grading (e.g. bulbus rete pegs, loss of epithelial cohesion) and malignant transformation. Similarly, Gilvetti *et al*. [31] demonstrated the importance of various clinical features, including age, in predicting outcomes for OED. Our study also found age to be a significant prognostic factor in one of the subgroups (0–50) with a *p* value of 0.001, corroborating the findings of Gilvetti *et al*. [31]. Our multivariate analysis, when we combined pathological and nuclear features, improved prediction of PFS, specifically due to the addition of epithelium layer NC and PELs count. An interesting avenue in future would be to analyse and investigate the association of dysplasia infiltrating lymphocytes (DILs) in malignant transformation. Although our cohort is small and unicentric, the department is a regional and national referral centre in the UK. Nonetheless, the practical application and adaptation of these methods in clinical practice will require substantially larger and multi‐center cohort data to allow for a more rigorous validation of the proposed algorithms.

To the best of our knowledge, this is the first study to propose and show the association of PELs count in malignant transformation along with other digital biomarkers, e.g. epithelium layer NC and basal layer NC. Our multivariate feature analysis showed that PELs and epithelial NC improve their prognostic value in conjunction with binary OED grading for predicting malignant transformation. Our proposed methodology for predicting malignancy has the potential to play an important role in precision medicine and personalised patient management for early prediction of malignancy risk with the potential to guide treatment decisions and risk stratification.

## Author contributions statement

RMSB, SEAR, SAK and NMR designed the study with the help of all co‐authors. RMSB, SEAR and NMR developed the computational methods. RMSB wrote the code and carried out all the experiments. SAK and HM obtained the ethical approval and retrieved the histological and clinical data. SAK, HM, AS, RMSB and NA provided the WSI annotations. RMSB, SEAR, SAK and NMR were all involved in the drafting of the paper. HM, AS and NA provided peer review for the final draft. SEAR and NMR jointly supervised the algorithmic development. All authors read and approved the final paper.

## Data Availability

All the data derived from this study are included in the manuscript. Source code can be made available, subject to intellectual property constraints, by contacting the last author (n.m.rajpoot@warwick.ac.uk).
